# Is Collective Efficacy Age Graded? The Development and Evaluation of a New Measure of Collective Efficacy for Older Adults

**DOI:** 10.1155/2012/360254

**Published:** 2012-01-23

**Authors:** Adena M. Galinsky, Kathleen A. Cagney, Christopher R. Browning

**Affiliations:** ^1^Center on the Demography and Economics of Aging, NORC and the University of Chicago, 1155 E. 60th Street, Chicago, IL 60637, USA; ^2^Departments of Sociology and Health Studies, University of Chicago, 1155 E. 60th Street, Rm 238, Chicago, IL 60637, USA; ^3^Department of Sociology, Ohio State University, 214 Townshend Hall, 1885 Neil Avenue Mall, Columbus, OH 43210, USA

## Abstract

*Objectives*. Community processes are key determinants of older adults' ability to age in place, but existing scales measuring these constructs may not provide accurate, unbiased measurements among older adults because they were designed with the concerns of child-rearing respondents in mind. This study examines the properties of a new theory-based measure of collective efficacy (CE) that accounts for the perspectives of older residents. *Methods*. Data come from the population-based Chicago Neighborhood Organization, Aging and Health study (*N = 1,151*), which surveyed adults aged 65 to 95. Using descriptive statistics, correlations, and factor analysis, we explored the acceptability, reliability, and validity of the new measure. *Results*. Principal component analysis indicated that the new scale measures a single latent factor. It had good internal consistency reliability, was highly correlated with the original scale, and was similarly associated with neighborhood exchange and disorder, self-rated health, mobility, and loneliness. The new scale also showed less age-differentiated nonresponse compared to the original scale. *Discussion*. The older adult CE scale has reliability and validity equivalent to that of the existing measure but benefits from a more developed theoretical grounding and reduced likelihood of age-related differential nonresponse.

## 1. Introduction

Evidence suggests that community processes are important to older adults' ability to age in place [1, 2]. Of the eight factors identified in the World Health Organization's report on age-friendly cities [3], three seem fundamentally dependent on community processes. These three, *Age-Friendly Outdoor Spaces* (WHO factor 1), *Social Participation* (WHO factor 4), and *Respect and Social Inclusion* (WHO factor 5) may all be supported by structural innovations and resource infusion, but, in all likelihood, cannot be sustained without on-going community involvement. Community-level behavior is important not only for the immediate results produced by discrete actions and social exchange, but also for its role in shaping the perceptions and norms of behavior held by the community's residents.

The perceptions and norms of behavior likely relevant to the three WHO factors fall under the rubric of a well-developed sociological construct, *collective efficacy*. Collective efficacy (CE) refers to perceptions and norms of two categories of social processes that represent two kinds of community social resources: trust and connection, commonly referred to as social cohesion, and expectations for action, commonly referred to as informal social control. Studies have shown the importance of CE for multiple aspects of well-being among older adults [4–7]. In particular, CE has been shown to play a role in enhancing older adults' physical health and neighborhood satisfaction, which may predict their intentions to move and actual migration [2, 5, 8–14]. Unfortunately, existing scales measuring this construct may not be ideal for use with older adults because they were designed with the concerns of child-rearing respondents in mind [15]. For example, scale items that ask about expectations of neighbor cooperation in monitoring children may be less relevant to adults whose children are grown. At the same time, the priorities of older adults are not necessarily reflected in these existing scales.

At the individual level, a number of scales measuring such constructs as anxiety and life satisfaction have been developed based on theory and evidence regarding the distinctiveness of older adults' experiences (e.g., [16–19]). These and similar scales are able to measure the constructs of interest among older adults more accurately and with less response bias because they take into account the unique concerns, challenges, and goals of adults in the later decades of their lives [20]. For example, scales that feature items that are more salient to older adults show increased instrument acceptability in the form of higher response rates and lower differential nonresponse [21]. Such scales, by providing more easily recognized and comprehended items, also reduce response burden [22]. By following the same principles, scales measuring neighborhood social processes can be designed such that they produce more accurate measurement among older adults.

In this study we describe and test a new measure of CE. This measure was developed specifically for use in older populations, taking into account the unique ways that people of their age and cohort interpret and respond to common environmental cues, and the particular cues that we hypothesized would be uniquely important to older adults. In the first part of this paper, we explain the theoretical framework guiding our identification of environmental cues for CE likely to be salient to older adults. In the second part of the paper, we test the new measure's instrument acceptability, dimensionality, reliability, and criterion validity in an older adult population. In the third part of the paper, we appraise the new measure's construct validity by examining its association with individual health-related outcomes. Our aim was to construct a scale that can be used in research on neighborhood social processes, the health of older adults, and other factors that relate to aging in place.

 Our theoretical framework combines CE theory with a consideration of the particular challenges and opportunities of the older adult life stage. As alluded to above, CE theory attempts to explain the association between neighborhood structural factors, social processes, and individual-level outcomes by positing that the neighborhood processes of social cohesion and informal social control mediate the relationship between the structural factors and individual outcomes [23, 24]. For example, compositional socioeconomic status may impact social cohesion, which in turn affects self-rated health, asthma rates, and inflammatory marker levels by reducing stress and fear [4–7, 25]. CE is therefore likely to be a part of the societal system that supports healthy living, safeguards individuals against adverse health events, and thereby enables aging in place [26]. CE is related to, but distinct from, social network interaction and exchange and social and physical disorder. The first is concerned with norms and expectations, while the second and third refer to actual behavior and conditions.

 A range of theories from the aging and life course literature provide us with a framework for generating a set of cues for social cohesion and informal social control that would be particularly salient to older adults [27–33]. A key focus of later life is to develop mechanisms to adapt to new challenges, including frailty and morbidity and decreased scope and density of social networks [30, 32, 34–37]. As applied to the CE framework, these perspectives suggest that perceptions of neighbors' willingness to assist older adults with tasks, and perceptions of neighborhood norms related to regulating behavior with the goal of enhancing neighborhood safety and traversability, will be particularly important. At the same time, older adults are not only concerned with compensating for losses and coping with challenges. Generativity is also a key component of later life, defined as helping the next generation by, for instance, passing on wisdom and thereby leaving a legacy [27–29]. Within the CE framework, this perspective incorporates the notion that intergenerational exchange may contribute to a prosocial orientation and a mutual respect for community contributions across the life course.

 In the remainder of this section, we discuss the research literature underlying our selection of the four specific types of cues for CE that we believe would be particularly salient to older adults. The two types of social cohesion cues that we hypothesize to be particularly salient to older adults, based on theory in urban sociology and literature on aging, are those that relate to *active caretaking of vulnerable residents* and *age integration/lack of ageism. *The two types of informal social control cues that we hypothesize to be particularly salient to older adults, based on the theory and literature on aging, are those that relate to *minimizing social incivility* and *maximizing accessibility*.

 Older adults may be particularly attuned to displays of solidarity in the form of social cohesion cues related to active caring and caretaking. Frailty and decreased mobility make some tasks that are easy in middle age significantly more difficult in later life [38, 39]. Simultaneously, many older adults experience a decrease in the scope and density of their social networks [40, 41]. As a result of this combination of changes, older adults are often more reliant on assistance from community members [42, 43]. Perceptions of the availability of neighbor assistance may be particularly important to the well-being of older women compared to older men and older single men compared to older married men, who are unlikely and unable, respectively, to rely on their spouses for help [44]. Older adults who believe that their neighbors will provide active caretaking may be more confident about their ability to stay in their homes. Our new measure of CE includes two items designed to capture the tendency toward active caretaking facet of the social cohesion construct.

 Older adults may also be particularly attuned to, and able to benefit substantially from, social cohesions cues related to age integration and lack of ageism. Aging societies have experienced an increase in social separation of age groups, even as age heterogeneity within most neighborhoods has increased [45–48]. One reason for the persistence of social segregation by age despite decreased logistical barriers to socializing across age boundaries may be ageism, which may interfere with communication across agelines [49]. Another factor impeding such communication is the decrease in information processing speed and loss of hearing that commonly occurs at older ages [50–52]. These factors combine to create a situation in which sustained effort is required for cross-generational socializing.

 Perceptions of opportunities for cross-generational interactions are relevant to older adults priorities, and ability to age in place, for a number of reasons. Communities in which older and younger people associate may be communities in which there are fewer age-based misunderstandings, biases, fears, and resentments, and greater empathy on the part of younger people for the challenges that come with later life [45, 48, 49, 53]. Older adults who perceive their communities as age integrated may therefore feel safer venturing outside to participate in community life, because they would have less reason to fear and more reason to feel connected to a wider range of their neighbors. Expectations of communication across age lines also encompass expectations for the ability to potentially pass on wisdom, neighborhood history, or practical advice and thereby feel and be useful [54]. Such opportunities for generativity are likely to be crucial to neighborhood satisfaction; being able to fill this social role has been associated with lower mortality [55]. Our new measure of CE includes three items designed to capture the age integration facet of the social cohesion construct.

 Regarding the domain of informal social control, we expect that older adults pay increased attention to the community's expectations for behaviors that minimize social incivility, as a result of reverse ageism (prejudice of older people against younger people), the increase in frailty that often accompanies advancing age [48, 56]. Older people with impaired balance, reduced muscle strength, and limited gait speed may be more likely than spry younger adults to find the loud, unpredictable peregrinations of rowdy teenagers threatening [48]. Furthermore, as a result of reverse ageism, elderly residents may perceive even subdued teenagers as a threat [56]. Their perceptions of the neighborhood's expectations for protecting vulnerable residents from malicious young people may be particularly important for older residents' willingness to venture outside on a regular basis and for their neighborhood satisfaction. Our new measure of CE includes one item designed to capture the expectation for actions that minimize social incivility, a component of the informal social control construct.

 Lastly, increased frailty and disability may also increase the salience of cues for informal social control in the form of expectations for behaviors that maximize accessibility. For many older adults, navigating their neighborhoods becomes more difficult as their mobility decreases and their vulnerability for adverse health outcomes resulting from interactions with environmental hazards increases [57–60]. Older frail adults may be more attuned to obstacles and hazards in the physical environment, particularly as those environments become dilapidated [61]. Increased expectations for actions aimed at improving the safety and integrity of the environment may be related to increased likelihood of maintaining and using physical abilities and competencies for two reasons: (1) it may be related to increased confidence about venturing outside, and (2) it may relate to actual improved conditions [8, 61, 62]. Maintenance of the environment can in turn prevent the adverse health events that constitute barriers to aging in place [13, 14, 26]. Our new measure of CE includes two items designed to capture the expectations for actions that maximize accessibility, a component of the informal social control construct.

## 2. Methods

### 2.1. Data and Sample

We used data from the Chicago Neighborhood, Organization, Aging and Health study (NOAH). This study surveyed 1,500 adults aged 65 and over living in 80 selected Chicago neighborhood clusters. Each cluster was defined by two to three census tracts consisting of approximately 4,000 housing units. The sample frame consisted of all households in the city of Chicago containing at least one member 65 years of age or older. The weighted response rate for households with a phone number was 55.3% while the rate for those households for which a phone number could not be identified was 12.4%. The overall weighted response rate for the survey was 44.3%, a good rate for a telephone interview by contemporary standards [63, 64]. Interviews were conducted over the phone in English and Spanish between August 2006 and September 2007. The NOAH study was approved by the institutional review boards of both NORC and the Division of Biological Sciences at the University of Chicago. All participants provided verbal consent.

 The sample consisted of the 76.7% of the 1,507 respondents with complete demographic, health, and community process data (*N* = 1, 151). Descriptive statistics are shown in Table 1. The mean age was 73 (range 65–95), and 68% were female. Over two fifths (44%) were non-Hispanic White, over a third were Non-Hispanic Black (35.8%), and the rest were Hispanic (14.9%) or Other (5%). About a third (32.6%) were married, and about a tenth (9.8%) lived with someone under the age of 18. About a quarter had less than 12 years of education, about another quarter had graduated from high school, and a little under half had some college or more. A little more than a tenth of the sample had lived in their neighborhood for less than 10 years, while a little less than three quarters had lived in their neighborhood for more than 20 years. Those missing data, who were therefore excluded from the sample as described above, were more likely to be White Non-Hispanic than to be any other race/ethnicity combination.

### 2.2. Measures of CE

The CE questions were presented to the respondent in two blocks. The first block was introduced with the sentences, “Now I'm going to read some statements about things that people in your neighborhood may or may not do. For each of these statements, please tell me whether you strongly agree, somewhat agree, somewhat disagree, or strongly disagree.” The second block was introduced with the sentence, “For each of the following, please tell me if it is very likely, somewhat likely, somewhat unlikely, or very unlikely that people in your neighborhood would act in the following manner.”

#### 2.2.1. Collective Efficacy—Original Scale Items

The original CE scale, composed of eight items, first appeared in the Project on Human Development in Chicago Neighborhoods [65]. In the NOAH survey, the items were administered alongside the new CE items. The following three items were in the first block: this is a close knit neighborhood; people around here are willing to help their neighbors; people in this neighborhood can be trusted. The next five items were in the second block: your neighbors would break up a fight in front of your house in which someone was being threatened or beaten; your neighbors would do something about it if a group of neighborhood children were skipping school and hanging out on a street corner; your neighbors would do something about it if some children were spray-painting graffiti on a local building; neighborhood residents would organize to try to do something to keep the fire station open if because of budget cuts the fire station closest to your home was going to be closed down by the city; people in your neighborhood would scold a child who was showing disrespect to an adult.

#### 2.2.2. Collective Efficacy—New Scale Items

The new CE scale, composed of eight items, was created by two of the authors. The age integration facet of social cohesion was measured with three items in the first block: people in your neighborhood treat older people in this neighborhood with respect; younger adults and children generally know who the older people in the neighborhood are; older people in this neighborhood socialize with younger adults as well as people their own age. The answer options for each of these were strongly agree, somewhat agree, somewhat disagree, and strongly disagree. The active caretaking facet of social cohesion was measured with two items, one in each block: your neighbors would shop for groceries for you, if you were sick; people in your neighborhood would check on older or more vulnerable residents if there was a heat wave. The answer options for the first item were the same as those for the age integration items. The answer options for the second item were very likely, somewhat likely, somewhat unlikely, and very unlikely. The maximizing accessibility facet of informal social control was measured with two items in the second block: people in your neighborhood would help to keep the sidewalks and other public spaces clear if there was a snowstorm; people in the neighborhood would help to get the problem corrected, if there was a problem in the neighborhood that affected older adults, like crumbling sidewalks or unsafe parks. The answer options for both items ranged from very likely to very unlikely. The minimizing social incivility facet of informal social control was measured with a single item in the second block: neighborhood residents would intervene if an older person in your neighborhood was being threatened by a group of teenagers. The answer options for this item again ranged from very likely to very unlikely.

### 2.3. Demographic and Health Measures

Sociodemographic measures included age, race/ethnicity, and marital status. An indicator for the presence of a child under the age of 18 in the household of the respondent was constructed using the list generated by a household roster. Health was measured using a self-report measure that asked: overall, how would you rate your health in the past 4 weeks: excellent, very good, good, fair, poor, or very poor? We treated self-rated health as an ordinal categorical variable, collapsing the categories poor and very poor into one, because less than 2% of the sample answered “very poor”. A measure of mobility was constructed using two measures taken from the Health and Retirement Survey (2002) and two measures adapted from the California Health and Interview Survey (CHIS). The resulting ordinal variable had the following categories: has difficulty walking across a room, has difficulty walking one block, walks less than 10 minutes or more each week, walks 10 minutes or more once or a few times each week, walks 10 minutes or more daily, walks 10 minutes or more multiple times a day. Loneliness was measured using Hughes et al. [66] three-item scale. It has a range of 0 to 3 and a mean of 1.4.

### 2.4. Neighborhood Process Measures

Besides CE, NOAH measured two other neighborhood processes. Neighborhood disorder was measured with a four-item scale from the PHDCN and was introduced with the sentence, “I'm going to read a list of things that are problems in some neighborhoods. For each, please tell me how much of a problem it is in your neighborhood—a big problem, somewhat of a problem, or not a problem.” The four items asked about litter, graffiti, drug use and sale, and public drinking. The scale was reliable in this sample (Cronbach's alpha = 0.74) and had a range of 1 to 3, with a mean of 1.54. Neighborhood exchange was measured with a four-item scale from the PHDCN and was introduced with the sentence, “Now I am going to ask about some things you might do with people in your neighborhood. For each, please tell me if it happens often, sometimes, rarely or never.” The four items asked about doing favors, watching over homes of absent neighbors, asking for advice, and visiting. The scale was reliable in this sample (Cronbach's alpha = 0.75) and had a range of 1 to 4, with a mean of 2.8.

### 2.5. Analysis

In the first section of the analysis, the properties of the new CE scale were examined. Instrument acceptability and item salience were examined by comparing response rates and differential nonresponse for each item in the original and new CE scales. Next, the new scale was examined for dimensionality using principal component analysis. Because only one factor was identified, the next step was to estimate internal consistency reliability by calculating Cronbach's alpha for the new scale. Criterion validity of the new scale was tested by calculating correlations with the original CE scale. Convergent validity of the new scale was tested by calculating the correlations of the new CE scale with other NOAH measures of neighborhood processes.

In the second section of the analysis, the construct validity of the new CE scale was tested in a two-step process. First, we examined the correlations between the new scale and the health, mobility and loneliness measures, comparing the results to those from identical analyses using the original CE scale. Second, we examined whether the new scale can predict well-being more accurately in certain demographic subgroups by comparing the fit statistics of regressions estimated in those subgroups.

## 3. Results

### 3.1. New Collective Efficacy Scale: Instrument Acceptability

We first examined percentage missing for each of the items in the original and new CE scales. The items most likely to be missing in the original CE scale were “do something about kids skipping school” (4.4%) and “scold child for showing adult disrespect” (3.7%). The items most likely to be missing in the new CE scale were “younger people know older people” (5%) and “older people socialize with younger adults” (4.6%). The percentage missing one or more item from the original CE scale was 10.9%, while the percentage missing one or more item from the new CE scale was 13.0%. No clear pattern emerged of one scale showing more missing than the other.

### 3.2. New Collective Efficacy Scale: Differential Nonresponse

The results of the differential nonresponse analysis are shown in Table 2. Consistent with previous research, respondents in the middle and oldest age categories were more likely than those in the youngest age category to be missing at least one item from both the original and older adult CE scales [22]. The extent of this differential nonresponse by age was not equal between scales, however. Those in the oldest age category were more likely to be missing *five* of the eight items in the old CE scale: the trustworthy neighbors item (7.9% versus 1.4%, *P* < 0.01), the scold a disrespectful child item (9.4% versus 2.4%, *P* < 0.01), the graffiti item (7.1% versus 1.7%, *P* < 0.05), the skipping school item (9.4% versus 3.1%, *P* < 0.05), and the break up a fight item (4.7% versus 0.7%, *P* < 0.05). In comparison, those in the oldest age category were only more likely to be missing *three* of the eight items in the new CE scale: the young people know older people item (11.0% versus 3.7%, *P* < 0.05), the older people socialize with young adults item (9.4% versus 3.7%, *P* < 0.05), and the neighbors intervene to protect threatened elder item (6.3% versus 1.7%, *P* < 0.05). Those in the middle age category did not differ from those in the youngest age category in their likelihood of missing any of the items in the new scale but did differ in their likelihood of missing one item in the original scale: the scold a disrespectful child item (4.9% versus 2.4%, *P* < 0.05). Because such a small percentage of the respondents were in the oldest age category (9%), we recalculated these percentages comparing the youngest old to the two older groups combined. In this case, those in the middle and oldest age categories were more likely to be missing four of the items from the original scale, but were only more likely to be missing two of the items from the new scale. In the old scale, these items were the trustworthy neighbors item (4.2% versus 1.4%, *P* < 0.01), the fire station item (3.7% versus 1.8%), the scold a disrespectful child item (5.8% versus 2.3%, *P* < 0.01), and the do something about a child skipping school item (6.3% versus 3.1%, *P* < 0.01). In the new scale, these items were the young people know older people item (7.0% versus 3.7%, *P* < 0.01) and the older people socialize with young adults item (6.0% versus 3.7%, *P* < 0.05).

### 3.3. New Collective Efficacy Scale: Dimensionality, Reliability, Criterion and Convergent Validity

The results from the principal component analysis suggested that the eight items in the new CE scale represented a single latent factor, since only one component had an eigenvalue greater than one. The internal consistency reliability of the scale, as measured by Cronbach's alpha, was 0.81. It was slightly higher for those over 77 and men (0.82 for both groups) and slightly lower for those 65–69 and women (0.79 and 0.80, resp.). The internal consistency reliability of the theoretically defined subscales was 0.65 (informal social control) and 0.72 (social cohesion).

 To examine the criterion validity of the new scale, we calculated its correlation with the old CE scale. The correlation of the scales with each other was 0.81, the correlation of the theoretically defined old and new social cohesion subscales was 0.68, and the correlation of the theoretically defined old and new informal social control subscales was 0.72.

We next tested for convergent and divergent validity by examining the association of the new CE scale with the two other NOAH measures of neighborhood quality, neighborhood disorder and neighborhood exchange (Table 3). Neighborhood exchange was more highly correlated with the new CE scale than it was with the original scale, while neighborhood disorder was more highly (negatively) correlated with the original CE scale than the new CE scale. Also, disorder was more highly (negatively) correlated with the new theoretically defined CE subscale of informal control than with the new theoretically defined CE subscale of social cohesion, while the reverse was true for exchange.

### 3.4. New Collective Efficacy Scale: Construct Validity

In the second part of the analysis, we examined the construct validity of the new scale by comparing its correlation with various health measures with similar correlations between the original scale and those measures. The correlations between the original and the new CE scales and the self-rated health, mobility, and loneliness measures are shown in Table 4. The correlation between self-rated health and CE, whether measured with the original or the new scale, was −0.17 (*P* < 0.0001). The correlation between mobility and CE, whether measured with the original or the new scale, was 0.07 (*P* < 0.05). The correlations between loneliness and the original and new CE scales differed. The correlation with the original scale was −0.16 (*P* < 0.0001) while the correlation with the new scale was −0.20 (*P* < 0.0001).

Lastly, we regressed each of these three measures on the two CE measures one at a time and compared the fit statistics. The fit of the models, as measured by the r-squared statistic, was not better for one scale than for the other (not shown). We also compared the fit statistics of these models estimated for the male and female subsamples, the married and unmarried subsamples, each of the age subgroups, and the sub-samples with and without children in their households (not shown). There were no differences in fit.

## 4. Discussion

The aim of this paper was to describe the development and examine the properties of a new theory-based measure of CE that incorporates the perspectives of older residents. One motivation for creating a new scale customized for a particular subpopulation is that the increased instrument acceptability and salience of the customized items may increase the response rate for the scale items. The results from our examination of the percentage missing the individual items, as well as percentage missing one or more items from the old versus the new CE scales, did not show such effects. Neither at the individual item level, nor at the scale level, did it appear that one scale is less or more likely to have missing values.

However, another motivation for creating a new scale customized for a particular subpopulation is that the increased salience of the customized items may decrease or eliminate differential nonresponse by the variables that define the subpopulation. The results from our analysis suggest such an effect in our new CE scale. The likelihood of missing was greater among the oldest old than the rest of the sample for five of the eight items in the original scale, but only three of the eight items in the new scale. The new scale is therefore better suited for use in older populations, because nonresponse will be less likely to be a function of age. This pattern was also in line with our hypothesis that items related to children would be less relevant and therefore harder to answer for older adults. Of the items with differential nonresponse, three of the five from the original scale and one of the three from the new older adult scale concerned young people.

The results of our dimensionality and reliability analysis suggest that the scale measures one factor, with good reliability. It has reasonable criterion validity, in that it was closely correlated with the original CE scale and the other two neighborhood scales, neighborhood exchange and disorder. It was perhaps to be expected that neighborhood exchange would be more highly correlated with the new CE scale than with the original scale, while neighborhood disorder would be more highly (negatively) correlated with the original CE scale than with the new CE scale, since the original CE scale has an equal number of social cohesion and informal social control items, while the new CE scale has 5 social cohesion items but only 3 informal social control items. This difference in the number of items measuring each part of CE may also explain why disorder was more highly (negatively) correlated with the new theoretically defined CE subscale of informal control than with the new theoretically defined CE subscale of social cohesion, while the reverse was true for exchange.

The results of the construct validity analysis suggest that the new CE scale predicts health and mobility just as well as does the original CE scale and may predict loneliness slightly better—an important finding given recent literature on the prevalence and salience of loneliness among older adults.

### 4.1. Limitations

The primary limitation of this study is its geographic specificity. Because it is limited to a single city, replication studies will need to examine the measure's psychometric properties in rural and suburban contexts, as well as in other urban areas. The other limitations of this study relate to its survey modality. Phone surveys are subject to sampling and response bias, the first exacerbated by increased use of call screening technology and the rapid growth of telephone marketing [64, 67]. However, the risk of social desirability bias inherent in the telephone survey administration modality should be relatively minor given the non-personal and therefore non-sensitive nature of most of the questions asked [68, 69]. Also, sampling or response bias due to hearing impairment is likely to be less significant than such bias due to vision and fine motor impairment in studies using self-administered questionnaires [22, 70].

## 5. Conclusions

The importance of neighborhood context, and in particular its potential ability to modify adverse health event risk, prevalence, and severity is being increasingly recognized. For example, the original measure of CE has just recently been added to the PhenX toolkit, a set of consensus measures intended to standardize genetic and epidemiological research (http://www.phenxtoolkit.org/-February 4 2011, Version 4.2). While the benefits to using standard measures include comparability across studies and the potential to easily combine results in meta-analyses, there are also benefits to using measures customized to particular populations. The new measure of CE presented in this study has reliability and validity equivalent to that of the existing measure but benefits from a stronger gerontology-related theoretical grounding and reduced likelihood of age-related differential nonresponse.

 The two measures exhibited both high correlation and comparable effects on the health outcomes considered. These findings raise the larger question of the extent to which measures of distinct forms of CE are capturing an underlying latent neighborhood capacity.

CE theory underscores the goal-directed nature of mobilization capacity, suggesting that a given neighborhood may have differing levels of CE depending upon the specific challenge under consideration. In this view, communities with high levels of CE with respect to the social control of public space may or may not share a comparable willingness to maintain and promote the health and well-being of local older adults.

 Yet, in practice, evidence suggests that high levels of CE across multiple objectives are likely to cluster together in the same communities. This may be due to the shared origins of distinct forms of CE in the structural (e.g., economic advantage, residential stability) and social (e.g., informal network density, voluntary organization participation) conditions of urban neighborhoods. Cohesive neighborhoods with high levels of mutual trust and solidarity may provide the conditions under which generalized prosocial norms emerge, benefiting a broad base of residential constituencies.

 Although the current analysis offers evidence consistent with the notion of a generalized collective capacity, we do not view these results as grounds upon which we reject the hypothesis that CE exhibits distinct dimensions. First, CE with respect to the social control of public space may have indirect benefits for older adults. Fear and the associated withdrawal from neighborhood environments may have important health implications for older adults and may be strongly related to local norms regarding the social control of children (a significant component of the original CE scale). Thus it may be the case that the original CE operates, in part, indirectly to produce comparable associations with the health outcomes considered. Second, research on the dynamics of neighborhood collective capacities is incipient. Analyses of the association and impact of CE measures focused on other shared goals (e.g., expectations regarding influence of local institutions) may reveal different patterns, warranting more extensive research.

In the case of older adult's perceptions of their communities' association with the factors that predict whether and how older adults age in place, the strength and mechanisms are still not fully understood. For example, it may be that even before health deteriorates, specific expectations that neighbors will provide help when needed and will take steps to maintain the safety of the common areas are the particular perceptions that predict intentions to stay. Similarly, it may be that when health and functionality deteriorate, the specific perception that neighbors are assuming the caretaking role usually shouldered by family may be the particular perception that forestalls a move. This new measure of CE can be used to test these hypothesized pathways, as well as the others discussed in the introduction (Section 1), that may link CE to neighborhood satisfaction, health, and the other factors that predict intention to move and actual migration.

## Figures and Tables

**Table 1:** Descriptive statistics.

	%
Age (years)	
65–74	59.7
75–84	31.4
85–95	8.6
Sex	
Female	67.7
Male	32.3
Race/ethnicity	
White Non-Hispanic	44.0
Black Non-Hispanic	35.8
Hispanic	14.9
Other	5.0
Married	32.6
Live with child under age 18	9.8
Education, in years	
<12	24.5
12	26.7
>12	48.7
Years in neighborhood	
<10	11.3
10–19	16.8
20–29	12.6
30–39	19.2
40+	40.1

**Table 2:** Percent missing each collective efficacy xcale item, by age group.

	Young old 65–**7**4^a^ *N* = 60% of the sample	Middle old 75–84^b^ *N* = 31% of the sample	Oldest old 85–95^b^ *N* = 9% of the sample
*Original collective efficacy scale items*			
Close knit neighborhood	0.9	0.1	0.3
Trustworthy neighbors	1.4**	3.2+	7.9**
Neighbors help	1.3	1.3	2.4
Fire station	1.8*	3.4+	4.7
Scold a disrespectful child	2.4**	4.9*	9.4**
Do something about children spraying graffiti	1.7	1.9	7.1*
Do something about children skipping school	3.1**	5.5+	9.4*
Break up a fight	0.7+	1.3	4.7*
Missing one or more item from the original CE scale	8.3***	13.6**	19.5**
*New collective efficacy scale items*			
Respect for old people	1.0	1.2	2.4
Groceries when sick	2.0	2.1	2.4
Young people know older people	3.7**	5.9+	11.0*
Older people socialize with young adults	3.7*	5.1	9.4*
Neighbors intervene to protect threatened elder	1.7	1.1	6.3*
Neighbors help fix issue affecting older adults	1.5	1.7	5.5+
Neighbors check on elders during heat wave	2.2	2.1	3.1
Neighbors shovel snow	1.7	2.5	0.8
Missing one or more item from the new CE scale	10.9***	14.4+	21.9**

^a^% missing differs from % missing among middle/oldest old, **P < 0.01*P < 0.05 + *P* < 0.1.

^b^% missing differs from % missing among young old, **P < 0.01*P < 0.05 + *P* < 0.1.

**Table 3:** Correlations of collective efficacy scales and subscales with other neighborhood scales.

	Original CE scale	New CE scale	New CE scale—social cohesion subscale	New CE scale— informal social control subscale
Disorder	−0.39	−0.33	−0.28	−0.33
Exchange	0.43	0.53	0.52	0.41

All correlations are significant at *P* < 0.0001, except that between disorder and exchange, which is significant at *P* < 0.01.

**Table 4:** Correlations of collective efficacy scales with health and well-being.

	Original CE scale	New CE scale
Self-reported health	−0.17***	−0.17***
Mobility	0.07*	0.07*
Loneliness	−0.16***	−0.20***

***P < 0.001**P < 0.01*P < 0.05 + *P* < 0.1.
